# What does digitalization hold for the creation of real-world evidence?

**DOI:** 10.1093/rheumatology/kez068

**Published:** 2019-12-13

**Authors:** Huai Leng Pisaniello, William Gregory Dixon

**Affiliations:** 1 Arthritis Research UK Centre for Epidemiology, School of Biological Sciences, Faculty of Biology, Medicine and Health, University of Manchester, Manchester, UK; 2 Department of Medicine, University of Adelaide, Adelaide, South Australia, Australia

**Keywords:** real-world evidence, electronic health record, mobile app, accelerometers, digital data, unstructured data, data protection, osteoarthritis

## Abstract

Health-related information is increasingly being collected and stored digitally. These data, either structured or unstructured, are becoming the ubiquitous assets that might enable us to comprehensively map out a patient’s health journey from an asymptomatic state of wellness to disease onset and its trajectory. These new data could provide rich real-world evidence for better clinical care and research, if they can be accessed, linked and analyzed—all of which are possible. In this review, these opportunities will be explored through a case vignette of a patient with OA, followed by discussion on how this digitalized real-world evidence could best be utilized, as well as the challenges of data access, quality and maintaining public trust.

**Rheumatology key messages**

- The volume and breadth of digital data contributing to real-world evidence is expanding.
- Digital data will allow researchers to answer questions that cannot currently be addressed.
- Real-world digital health data require robust data governance, sustainable public trust, data standardization and interoperability.

## Introduction

The increased uptake of technology is changing our ability to observe and understand the onset, progression and outcome of disease in society. Information and communication is increasingly stored digitally. There has been an exponential expansion in stored data, from digital versions of traditional media like text, to images and videos, sensors, digital transactions and even digital traces of our interactions with technology [1, 2]. As we live our daily lives, vast amounts of information pertaining to our health and well-being are being recorded, including contact with health care systems. This includes our exposure to environmental and behavioral risk factors while living free from disease, the onset of symptoms and progress towards a clinical diagnosis, as well as the consequences and impact of living with a disease and its treatment. Digital data relevant to health are expanding from the more obvious and traditional, for example, notes held within a medical record, to the sometimes less apparent information captured about our everyday lives, as well as a wide array of data describing the environment in which we live. This digital archive of information is fragmented and scattered, sometimes unstructured, yet it has the potential to help us better understand diseases and their treatment and ultimately to improve the lives of future populations. This article will consider the wide range of data that exists, both traditional and novel, that might contribute to real-world evidence (RWE) about health and disease.

### Spot the digital data sources

Let us consider a patient’s health journey, starting from a pre-morbid state of wellness to the onset of disease symptoms, self-management and interactions with health care professionals, to treatment response and disease outcome. Through a working example, we will examine how a patient may seek help, information, support, guidance and treatment through this journey, with an eye on what digital data are captured.

Austin is a middle-aged man who has been previously fit and well, albeit slightly overweight. He enjoys running three times a week and tracks his activity, performance and heart rate using a heart rate monitor linked to his smartwatch, allowing him to understand his achievements and progression. He is in his late 40s, and he has begun to experience persistent knee pain. His family history of OA and discussions on his online fitness community site make him wonder whether he is developing arthritis. He seeks information to learn more about his symptoms through web searches and online forums about OA. He sees his general practitioner (GP) about his knee pain. A diagnosis of knee OA is confirmed and he is referred to a physiotherapist. He is given an exercise prescription, education on lifestyle modification and advice on simple analgesia. He buys over-the-counter (OTC) paracetamol and topical NSAID gel at his local supermarket. Follow-up visits are arranged to assess his knee OA progression and management. In the meantime, he continues to self-monitor and tries to identify possible triggers for days that are worse. He sends his saliva to a commercial company to generate a genetic health risk and wellness report. He experiments with nutritional supplements and alternative health food.

Over the next few years, he notes a progressive deterioration in his performance and an increase in pain. He is referred to an orthopaedic surgeon where he is assessed, has further input from the physiotherapist, has X-rays confirming disease progression and is given a dedicated smartphone application (mobile app) for his pre- and post-operative assessment. He agrees to undergo knee replacement surgery, but the outcome is not what he expected. Despite an initial good recovery, his knee pain persists, which is evident from his pain-tracking mobile app. Given his ongoing frustration with an outcome that did not meet his expectations, he becomes an avid blogger in sharing his post-surgical knee pain experience with others.

**Fig. 1:**
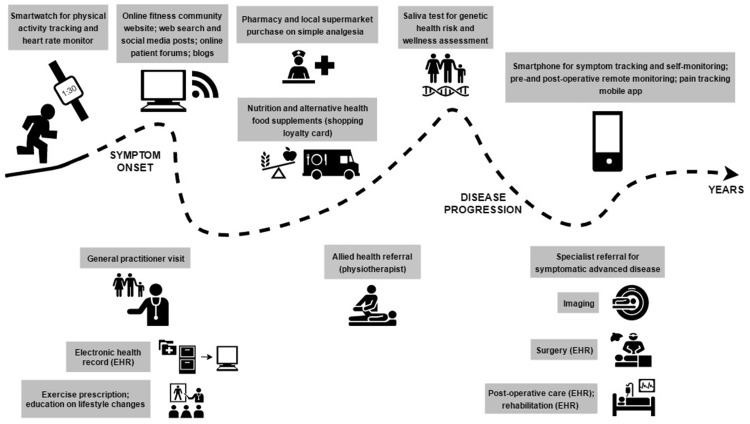
Digital health data capture: a hypothetical case study of the onset and progression of OA EHR: Electronic health record.

Throughout Austin’s journey, digital data are captured that could build a picture of his disease and its antecedents, treatments and outcome (Fig. 1). Each discrete data source would show part of his journey but, if linked, could show a more comprehensive picture. These data include what we might consider traditional health data: primary and secondary electronic health records (EHRs) from his GP, specialists and allied health professionals, as well as imaging data. The clinician-derived data are supplemented by patient-generated health data within the health care system, such as his peri-operative mobile app.

There are also recorded data from the patient outside of clinical encounters, including his wearable tracking record, web search history, supermarket loyalty card, genetic profile and social media posts. Collectively these longitudinal types of digital data can provide real-time tracking of symptom trajectory and disease progression and outcome. This opportunity will support individualized and evidence-based understanding of our patients, not only in terms of disease impact on their general well-being, but also of their digitalized information-seeking behavior. Analyses of observational data from some of these sources, such as primary care EHRs, already contribute to our understanding of disease and RWE [3–5]. There are many others, like sensors and social media posts, that offer potential RWE, although experience of how to access and analyze such data is, at present, limited.

Observational data in knee OA have indeed added considerably to our knowledge. Age is one of the strongest risk factors for knee OA, perhaps because of an accumulation of other risk factors with time coupled in with the aging process and a reduced ability to withstand adversity in the joint [6]. Obesity is another well-established local risk factor for knee OA [7–9]. Existing knowledge about OA risk factors is often restricted to insights derived from data that are routinely collected, such as age, gender, weight, smoking status, family history and other comorbidities [9]. There is a paucity of evidence about some less readily available risk factors, such as physical activity, diet, other lifestyle factors and health-seeking behavioral, in influencing the development and progression of knee OA. Physical activity is a putative risk factor for knee OA, yet studies provide conflicting evidence [10–12]. This may be due in part to the challenge of accurately measuring and summarizing physical activity patterns over many years before the onset of disease. Prior studies examining this question have used crude measures such as a single self-reported physical activity questionnaire or by comparing, say, runners to non-runners [13, 14]. Yet, as seen in Austin’s case study, people are leaving behind them very detailed, digital traces of their active and sedentary living. If these can be linked to health and disease onset and outcomes, we might significantly advance our learning about the risk factors for the onset of knee OA.

Let us now consider Austin’s surgery. In Austin’s case, why did his total knee replacement (TKR) have a worse outcome compared with similar patients? Many observational studies have examined predictors of poor outcome in TKR. Studies have previously identified factors such as higher pre-operative pain and functional limitations, social disadvantage, depression and anxiety, higher fatigue and higher illness-related distress and co-existing medical conditions [15–17]. Yet Austin had good mental health, high socio-economic status and few comorbidities—so why him? It is possible that he had other important predictors of a poor outcome that may not be easily identified through these study designs. These might include issues such as the timing of surgery with respect to his functional deterioration or his post-operative exercise and other relevant activities during his surgical recovery and rehabilitation. Such metrics and data were collected within his personal digital history but are not yet commonly analyzed across large populations.

As well as contributing to population-level RWE, Austin’s use of technology might also support timely interventions. When Austin is faced with intractable pain post-TKR, might his pain be better managed if his day-to-day pain-tracking data, medication use and physical activity data could be accessed? These data might allow his treatment to be personalized instead of escalating the dose and strength of his analgesia and giving generic advice on exercise and self-care. Consumer devices such as activity trackers already employ smart coaching techniques to encourage greater physical activity, guided by contemporaneous data collected on the device. In time, it is possible that real-time analysis of this RWE could lead to personalized digital health interventions, such as post-operative coaching, to support usual clinical care.

## Challenges in providing digitalized RWE in future health care and research

### New data types

Research that uses the novel data sources described in Austin’s case study is still in its infancy. Studies in knee OA are starting to use sensors to evaluate mobility, demonstrating this method of assessment is feasible and may be cost-effective [18–20]. Longitudinal studies using accelerometers are starting to collect much more granular information about physical activity in patients with knee OA, such as the frequency, intensity, time and type of activity [21]. Many such studies provide participants with a research device to track their activity but do not yet provide RWE in free-living individuals without an associated research infrastructure. Although large-scale bespoke research studies using loaned accelerometers can be done, as seen in the UK Biobank study, which provided wrist-worn devices to >100 000 participants, such efforts are a major undertaking [22]. Research using physical activity measured using consumers’ own devices is starting to emerge—and sometimes on a large scale. Using data from the Argus app by Azumio (Palo Alto, CA, USA), researchers compared physical activity levels in 717 527 people from 111 countries across the globe [23].

Despite offering big promise, there are open questions such as the validity and quality of the activity measurement and possible selection bias of smartphone and app users. In studies comparing step counts from consumer wearables and smartphones in healthy adults, variability in step count accuracy has been seen between devices [24]. In knee OA specifically, small feasibility studies are exploring whether patterns of physical activity can be collected using raw accelerometer outputs alongside self-reported data using consumer cellular smartwatches [25]. This would make the derivation of a physical activity metric more transparent and standardized and could potentially lead to a future where we are able to have detailed daily information about disease symptoms and progression collected on a single device. The gradual introduction of patient-generated health data from consumer devices into clinical care will lead to significant opportunities for research due to the additional non-clinical context and information available from linkable clinical records data. The overlap between what data and information could, in theory, support both clinical care and research is significant, meaning careful design of systems to meet both needs would deliver multiple benefits [26, 27].

The science of analysing user-generated data from web searches and social media posts is new but has great potential [28]. There are already examples of both promising insight and notable errors from this data mining approach. One of the most highly cited examples of web data analytics was the Google Flu Trends service, which was initially heralded as an exemplary use of big data but was later found to generate inaccurate predictions [29, 30]. Other areas of social media mining for health beyond disease surveillance have included pharmacovigilance and behavioral medicine [31]. Both of these areas have relevance to our case study. Could analysis of OTC NSAID use, captured through store card data or self-reported information, tell us about its efficacy, or could studying paracetamol consumption shine further light on controversies about its effectiveness and safety [32]? Paracetamol safety is notoriously difficult to study using existing data sources such as administrative databases or primary care databases, because they do not capture OTC use that accounts for the vast majority of paracetamol use. As discussed above, how does physical activity influence the onset and outcome of OA? There is an increasing range of research that has explored unstructured data obtained from social media platforms in different rheumatic and musculoskeletal conditions [33–40]. For example, analysis of gout-related social media posts has shown patients are more interested in symptom uncertainties and treatment and less so in serological results of urate and its treat-to-target level [33]. Other studies using social media have brought to light patients’ concerns about treatment, for example about biologics or prednisolone therapy [35, 41, 42]. Patients can find it difficult to discuss certain views openly with their clinicians, so analysis of their views captured digitally outside of the clinic consultation can be insightful.

### Fundamentals of epidemiology for a digital age: selection bias, validity, missing data and more

The new world of digital health data to support observational research requires us to revisit several fundamentals of epidemiology. Selection bias can be easily introduced, as digital health studies may recruit specific types of participants, such as individuals who are health conscious, digitally literate or have a higher socio-economic class. The validity of new data collection tools needs to be considered, whether it is a digital version of a traditional measure such as a visual analogue scale, a new instrument for self-reported data, an active task such as an app-directed 6-minute walk test or raw or processed passively collected sensor output. For example, can we trust range of motion as measured by a user holding a smartphone and following instructions on a mobile app [43]? Processing and analysis of such data require the establishment of new standards with transparent reporting. For unstructured data, there is a technical challenge in accurately converting these data into structured forms ready for population-level analysis. Researchers may frequently face issues of missing data and diminishing data over time, as a gradual downtrend of user engagement is commonly seen over time in many mobile health studies [43, 44]. Handling continuous streams of sensor data will require a new analysis method not previously required for many epidemiological studies. There have been enormous advances in artificial intelligence (AI) and machine learning in recent years. Progress in non-health industries, such as financial services, the development of driverless cars, speech recognition within smartphones and fraud detection in insurance, has been exceptionally rapid and fruitful. Similar progress is now starting to appear in health care [45, 46]. In OA, the use of AI in automated multidimensional imaging analysis may allow complex computational interpretation and aggregation of these sophisticated imaging data, linking them to patient-generated health data and clinical care data [47]. There are concerns that predictive analytics in health, despite good model performance, may not be sufficiently transparent to enable clinical buy-in and trust, a challenge that may be helped by emerging developments in ‘interpretable’ machine learning or ‘explainable AI’ [48].

### Governance and public trust for real-world digital health data

Each discrete data source described above and its associated analysis is promising, yet it is clear that the real value will come when different digital data sources can be collated to give a more comprehensive picture.

Appropriate governance on data ownership and data protection is imperative as we move towards the idea of acquiring, aggregating and archiving linked digital health data. Patients should be able to control their own data, with clarity about who has handled their data. This will allow them freedom and rights in protecting, linking and sharing their data with other digital health users, such as health care professionals and researchers. It is inevitable that only certain members of society would wish to have their data shared in this way for research, and their views should be heard. Yet this should not necessarily prevent any data sharing within society. Initiatives like CitizenMe allows individuals to store their digital data in their personal data cloud, as well as to participate in surveys which may provide small monetary incentives [49].

Official regulation in reinforcing data security, consent for data linkage and privacy is important in the digital era [50]. Standards and guidelines are emerging, but it remains a gray area at times. In the UK, the use of anonymized EHR data for research does not require patient consent [51]. In primary care research databases such as the UK’s Clinical Practice Research Datalink, the data controller does not hold identifiable patient information and therefore cannot facilitate contact with patients. Nonetheless, it is possible for studies to collect data from patients alongside their EHR data via their GP, albeit with limited uptake from practices and patients [52].

Keeping public trust can sometimes require more than abiding by governance regulations, and so researchers must be thoughtful about how they clearly communicate the benefits and managed risks of data sharing [53]. Analysis of social media data still requires care, as users may not understand that their data are publically available and may not wish their data to be used for research [54]. Future digital health and social care data require a bona fide and secure infrastructure for data storage and use. As outlined by Mandl and Kohane [55, 56], standardization and interoperability of different digital data sources are crucial for ensuring correct and valid data acquisition from patients and appropriate implementation of these data in self-care, clinical care and research. When collecting consent on digital devices, a new model of consent is required in the absence of study nurses, traditional consent forms and patient information sheets. Guidelines for electronic consent have been published by the US Food and Drug Administration, and there are already examples of excellent practice in mobile health studies such as the MyHeart Counts cardiovascular health study [44, 57].

While there remain lots of challenges in the area of governance, citizen consent and privacy are well delivered in many other aspects of our digital lives, such as banking, so strong governance of health data with public trust should be entirely possible. Recent initiatives such as the Wellcome Trust’s Understanding Patient Data, Health Data Research UK and the new Ada Lovelace Institute, an independent research and deliberative body with a mission to ensure data and AI work for people and society, will help ensure public trust remains at the forefront of developments in health data and research [53, 58]. As stated in the report from the Select Committee on Artificial Intelligence, ‘maintaining public trust over the safe and secure use of their data is paramount to the successful widespread deployment of AI and there is no better exemplar of this than personal health data’ [59].

## Conclusion

In summary, data about the causes and determinants of disease and its outcome are increasingly being collected digitally. It is already possible to see that such data will be hugely valuable. We are moving from a time when disease could be measured only at sparse intervals, such as at a 6-month clinic appointment, to a situation where many aspects and correlates of disease can be tracked frequently or for the first time. Novel data types provide an opportunity to answer questions that were previously difficult or impossible to answer. Yet there remain significant challenges around the appropriate governance of such data that maintains public trust and how we ensure we derive appropriate insight given the representativeness of the new digital patient. The inevitable move into the digital era means we should embrace, rather than hide behind, these challenges and ensure we make the best use of the opportunities that this new RWE presents to us.
